# Coexistence of Proteinase 3 (PR3)-Positive Granulomatosis With Polyangiitis and Genetically Confirmed Alport Syndrome in a 31-Year-Old Female Patient: A Diagnostic and Management Challenge

**DOI:** 10.7759/cureus.107982

**Published:** 2026-04-29

**Authors:** Laura Valdes, Angelica Ludena, Juan Carlos Pantoja Miranda, Joham Ortiz, Sohair Angly

**Affiliations:** 1 Department of Internal Medicine, Larkin Community Hospital, South Miami, USA

**Keywords:** alport syndrome, genetic test, granulomatosis with polyangiitis (gpa), hereditary diseases, pr3-anca

## Abstract

Distinguishing autoimmune glomerulonephritis from hereditary nephropathy is critical to avoid unnecessary immunosuppression and ensure appropriate long-term renal monitoring. A 31-year-old Hispanic female patient with a complex medical history, including proteinase 3 (PR3)-positive granulomatosis with polyangiitis (GPA), rheumatoid arthritis, Raynaud phenomenon, seizure disorder, autism spectrum disorder, bipolar disorder with psychotic features, and cochlear implantation in childhood, was found to have genetically confirmed Alport syndrome through Natera testing (Natera, Inc., Austin, Texas, United States). Despite preserved renal function (estimated glomerular filtration rate (eGFR) 107 mL/minute/1.73m²), urinalysis demonstrated persistent proteinuria. Her history of early-onset hearing loss requiring cochlear implantation raised suspicion for hereditary nephropathy. Elevated anti-PR3 antibodies were present without clinical evidence of active vasculitis. The coexistence of autoimmune disease and hereditary collagen IV nephropathy complicated the attribution of urinary abnormalities. This case highlights the importance of genetic evaluation in patients with multisystem disease and urinary abnormalities, particularly when clinical features such as early-onset sensorineural hearing loss are present. Recognition of Alport syndrome in patients with coexisting autoimmune vasculitis has significant implications for renal surveillance, immunosuppressive stewardship, and multidisciplinary management.

## Introduction

Alport syndrome is an inherited disorder of type IV collagen caused by pathogenic variants in the *COL4A3*, *COL4A4*, or *COL4A5* genes. It is characterized by persistent hematuria, progressive proteinuria, sensorineural hearing loss, and ocular abnormalities. Its prevalence is estimated to range from approximately one in 5,000 to one in 53,000 individuals worldwide, although this is likely underestimated due to underdiagnosis of milder phenotypes. The incidence is estimated at approximately one in 50,000 live births, and the condition accounts for 2-3% of pediatric chronic kidney disease and up to 0.2% of adult end-stage renal disease cases [1,2].

Renal involvement typically progresses to chronic kidney disease over time. Granulomatosis with polyangiitis (GPA) is a small-vessel vasculitis associated with antineutrophil cytoplasmic antibodies (ANCA), commonly directed against proteinase 3 (PR3). Renal manifestations can include rapidly progressive glomerulonephritis. The coexistence of hereditary nephropathy and ANCA-associated vasculitis is rare and presents diagnostic challenges, particularly when urinary abnormalities are present in the absence of overt renal dysfunction [3,4].

Although these conditions differ fundamentally in pathophysiology, hereditary structural defects versus immune-mediated inflammation, they may present with overlapping renal findings, particularly proteinuria and urinary abnormalities. This overlap can create diagnostic uncertainty, especially in patients with known autoimmune disease who develop persistent urinary abnormalities without clear evidence of active inflammation [1,2,5]. Distinguishing between hereditary nephropathy and inflammatory glomerular disease is critical, as misattribution of renal findings to active vasculitis may lead to unnecessary immunosuppressive therapy, while failure to recognize Alport syndrome may delay appropriate nephroprotective interventions and genetic counseling.

We present the case of a young woman with PR3-positive GPA and persistent proteinuria who was ultimately found to have genetically confirmed Alport syndrome. This case highlights the importance of integrating clinical, laboratory, and genetic data to accurately differentiate between overlapping renal pathologies and guide appropriate management.

## Case presentation

A 31-year-old Hispanic woman was evaluated via telehealth for routine follow-up. Her medical history was notable for PR3-positive GPA, rheumatoid arthritis, Raynaud phenomenon, mild persistent asthma, seizure disorder, autism spectrum disorder, intellectual disability, bipolar disorder with psychotic features, generalized anxiety disorder, gastroesophageal reflux disease, and recurrent urinary tract infections. She also had a history of cleft palate repair during childhood and right-sided cochlear implantation at approximately 10-11 years of age due to sensorineural hearing loss. Family history was notable for systemic lupus erythematosus, dermatomyositis, rheumatoid arthritis, and asthma in her mother.

Her home medications included: hydroxychloroquine 200 mg twice daily, amlodipine 5 mg daily, metformin extended-release 750 mg daily, valproic acid 500 mg twice daily, topiramate 200 mg twice daily, paroxetine 40 mg daily, risperidone 4 mg twice daily, benztropine 2 mg twice daily, alprazolam 1 mg up to four times daily as needed, fluticasone intranasal spray, albuterol inhaler as needed, and famotidine 20 mg daily; rituximab and methotrexate had been discontinued 10 months earlier due to medication refill issues.

Vital signs were stable with blood pressure 121/62 mmHg, heart rate 83 beats per minute, and temperature 97.2 °F. Body mass index (BMI) was approximately 44.6 kg/m² (prior weight 252 lb; height 5 ft 3 in). Notable physical findings included: mild saddle-nose deformity, bilateral knee tenderness without synovitis, dry hyperpigmented skin patches, stable neurological examination, mood was generally appropriate, though intermittent inappropriate laughter was observed.

Laboratory evaluation revealed a normal estimated glomerular filtration rate (eGFR) of 107 mL/minute/1.73 m², HbA1c of 6.1%, reduced vitamin D 25-hydroxy level of 10.2 ng/mL, and elevated anti-PR3 antibody levels of 2.8 (Table 1). Urinalysis demonstrated proteinuria of 180mg/day and leukocyte esterase positivity but no hematuria or nitrites. Lipid panel showed LDLc (low-density lipoprotein cholesterol) of 93 mg/dL.

**Table 1:** Laboratory test results MCV: mean corpuscular volume; eGFR: estimated glomerular filtration rate; HbA1c: glycated hemoglobin; PR3: proteinase 3; MPO: myeloperoxidase

Test	Patient Value	Reference Range	Interpretation
Hemoglobin	13 g/dL	12–16 g/dL	Normal
MCV	75 fL	80–100 fL	Microcytosis
Creatinine	0.76 mg/dL	0.6–1.1 mg/dL	Normal
eGFR	107 mL/min/1.73m²	>90	Normal renal function
HbA1c	6.1%	<5.7%	Prediabetes
Vitamin D (25-OH)	10.2 ng/mL	30–100 ng/mL	Deficient
Anti-PR3	2.8	<1.0	Elevated
Anti-MPO	Negative	Negative	Normal
Proteinuria	180 mg/day	<150 mg/day	Mildly elevated

Given the combination of early-onset sensorineural hearing loss and persistent proteinuria, a hereditary nephropathy was suspected. Genetic testing was performed through Natera (Natera, Inc., Austin, Texas, United States), which identified a *COL4A5* pathogenic variant consistent with Alport syndrome, establishing a diagnosis of a collagen IV-related disorder.

This diagnostic clarification was critical in distinguishing the cause of the patient’s urinary abnormalities. While GPA can cause inflammatory glomerulonephritis, it typically presents with hematuria, active urinary sediment, and declining renal function. In contrast, this patient’s stable renal function, absence of inflammatory urinary findings, and characteristic extrarenal manifestation (sensorineural hearing loss) supported a hereditary etiology of kidney disease rather than active vasculitic involvement.

Thus, the patient’s clinical picture represents the coexistence of two distinct but clinically overlapping conditions: autoimmune disease (GPA) and hereditary nephropathy (Alport syndrome). Recognizing this relationship allowed for appropriate management focused on renal protection rather than escalation of immunosuppressive therapy.

Table 2 summarizes the key clinical findings and diagnostic interpretations.

**Table 2:** Summary of key clinical findings and diagnostic interpretation eGFR: estimated glomerular filtration rate; SLE: systemic lupus erythematosus; RA: rheumatoid arthritis; ANCA: antineutrophil cytoplasmic antibodies; GPA: granulomatosis with polyangiitis

Category	Findings	Clinical Interpretation
Demographics	31-year-old Hispanic female patient	Young patient with multisystem disease
Relevant History	PR3-positive granulomatosis with polyangiitis (GPA), rheumatoid arthritis	Known autoimmune background increases suspicion for inflammatory renal disease
Key Clue	Early-onset sensorineural hearing loss requiring cochlear implantation	Strongly suggestive of hereditary nephropathy (Alport syndrome)
Family History	Autoimmune diseases (SLE, RA)	Supports autoimmune predisposition but not specific for hereditary nephropathy
Renal Function	Creatinine 0.76 mg/dL, eGFR 107 mL/min/1.73 m²	Preserved kidney function; argues against active vasculitic glomerulonephritis
Urinalysis	Persistent proteinuria, no hematuria, no active sediment	More consistent with hereditary nephropathy than inflammatory process
Serology	Elevated PR3 antibodies, MPO negative	Suggests GPA but does not confirm active disease
Genetic Testing	Pathogenic *COL4* mutation (Natera)	Confirms diagnosis of Alport syndrome
Inflammatory Features	No hematuria, no RBC casts, no renal decline	No evidence of active ANCA-associated renal involvement
Final Interpretation	Coexistence of GPA and Alport syndrome	Urinary findings attributed to hereditary nephropathy rather than vasculitis
Management Implication	Initiation of losartan, nephrology follow-up, no escalation of immunosuppression	Focus on renoprotection and avoidance of unnecessary immunosuppressive therapy

## Discussion

Alport syndrome is caused by pathogenic variants in the *COL4A3*, *COL4A4*, and *COL4A5* genes, which encode the α3, α4, and α5 chains of type IV collagen, a critical structural component of basement membranes in the kidney, cochlea, and eye. These collagen chains normally assemble into a triple helix that provides mechanical stability and filtration integrity to the glomerular basement membrane (GBM). Mutations in these genes disrupt normal collagen assembly, resulting in thinning, splitting, and progressive dysfunction of the GBM [1,2].

The disorder is most commonly inherited in an X-linked pattern due to *COL4A5* mutations, accounting for approximately 80% of cases, while autosomal recessive and autosomal dominant forms related to *COL4A3* and *COL4A4* mutations are less frequent. The phenotypic expression varies depending on the specific mutation and inheritance pattern, ranging from isolated hematuria to progressive chronic kidney disease leading to end-stage renal disease [5-7].

The clinical manifestations of Alport syndrome directly reflect the distribution of type IV collagen. In the kidney, structural abnormalities of the GBM lead to persistent hematuria and progressive proteinuria. In the cochlea, collagen defects contribute to sensorineural hearing loss, often presenting in childhood or early adulthood. Ocular manifestations, such as anterior lenticonus and retinal abnormalities, may also be present [1,2].

In this patient, the identification of a pathogenic *COL4* mutation provides a unifying explanation for the combination of early-onset sensorineural hearing loss and persistent proteinuria. Importantly, understanding the genetic basis of Alport syndrome has significant clinical implications. It allows clinicians to distinguish hereditary nephropathy from inflammatory renal processes, particularly in patients with coexisting autoimmune conditions. This distinction is essential, as misattributing urinary abnormalities to ANCA-associated vasculitis could lead to unnecessary immunosuppressive therapy and associated risks.

Furthermore, recognition of Alport syndrome supports the implementation of targeted management strategies, including early initiation of renin-angiotensin system blockade to reduce proteinuria and slow disease progression, as well as long-term nephrology follow-up [1]. It also has implications for genetic counseling and family screening. In this case, integrating genetic findings with clinical features was critical in guiding appropriate management and avoiding overtreatment.

This case illustrates the diagnostic complexity of urinary abnormalities in a patient with coexisting autoimmune vasculitis and hereditary nephropathy. ANCA-associated vasculitis frequently produces rapidly progressive glomerulonephritis characterized by hematuria, red blood cell casts, and declining renal function [3,4]. In contrast, this patient demonstrated preserved renal function and lacked hematuria or active urinary sediment, making active vasculitic renal involvement unlikely. Persistent proteinuria in the context of genetically confirmed Alport syndrome, therefore, represents a more plausible explanation for the urinary findings.

Several historical features support the diagnosis of Alport syndrome: early-onset sensorineural hearing loss requiring cochlear implantation, persistent urinary abnormalities, and genetic confirmation of a pathogenic collagen variant. Although granulomatosis with polyangiitis can affect the ear, it typically produces conductive hearing loss related to middle ear disease rather than primary sensorineural impairment [1,3].

Failure to recognize hereditary nephropathy in patients with autoimmune disease may lead clinicians to attribute urinary abnormalities to inflammatory renal pathology [8,9]. Such misattribution can result in unnecessary exposure to immunosuppressive therapy and its associated risks. Conversely, early recognition of Alport syndrome allows implementation of nephroprotective strategies aimed at slowing renal disease progression.

Management considerations following the diagnosis of Alport syndrome include regular monitoring of urine protein excretion, periodic assessment of renal function, consideration of renin-angiotensin system blockade if persistent proteinuria develops, and nephrology referral for long-term surveillance. In parallel, coordinated rheumatologic follow-up remains essential for management of the patient’s autoimmune conditions, particularly given the interruption of rituximab and methotrexate therapy [1]. Additional referrals to pulmonology and neurology were pending at the time of evaluation. 

Optimization of metabolic risk factors is also recommended, including weight reduction, vitamin D supplementation, and continued monitoring of glycemic status. These measures may help mitigate long-term renal and cardiovascular risk. 

This case has several limitations. Longitudinal data regarding the progression of proteinuria and long-term renal outcomes were limited at the time of evaluation. Detailed family genetic testing and pedigree analysis were not available, which may have provided further insight into inheritance patterns and disease expression. Furthermore, while genetic testing confirmed a pathogenic variant consistent with Alport syndrome, correlation with renal biopsy findings was not performed. Despite these limitations, this case highlights important diagnostic considerations in distinguishing hereditary nephropathy from inflammatory renal disease in patients with complex clinical presentations.

## Conclusions

This case illustrates the diagnostic complexity of evaluating urinary abnormalities in a patient with coexisting autoimmune and hereditary conditions. In this patient, the combination of persistent proteinuria, preserved renal function, absence of active urinary sediment, and early-onset sensorineural hearing loss, together with genetic confirmation of a pathogenic collagen IV variant, supports a hereditary etiology consistent with Alport syndrome as the most likely explanation for the renal findings. At the same time, the presence of PR3-ANCA positivity underscores the importance of careful clinical correlation, as serologic findings alone do not establish active vasculitic disease. The absence of features typically associated with ANCA-related glomerulonephritis, such as hematuria, red blood cell casts, and declining renal function, argues against active renal involvement at the time of evaluation.

However, given the lack of histopathologic confirmation and limited longitudinal follow-up, a cautious interpretation is warranted, and the possibility of future vasculitic renal involvement cannot be fully excluded. This highlights the need for ongoing surveillance and multidisciplinary management. Overall, this case emphasizes the importance of integrating clinical, laboratory, and genetic data to accurately differentiate overlapping renal pathologies. Such an approach is essential to avoid unnecessary immunosuppression while ensuring appropriate nephroprotective strategies and long-term monitoring.
